# Diapause Induction, Color Changes, and Supercooling Point of Diapause Larvae of *Tetrastichus septentrionalis* Yang (Hymenoptera: Eulophidae)

**DOI:** 10.3390/insects14100826

**Published:** 2023-10-20

**Authors:** Zhixin Li, Junrui Shi, Liyuan Yang, Yiran Cheng, Xudan Liu, Shouhui Sun

**Affiliations:** College of Forestry, Shenyang Agricultural University, Shenyang 110866, China; zhuti0225@163.com (Z.L.); aurora6800@163.com (J.S.); yangliyuan_good@126.com (L.Y.); czlhcl@gmail.com (Y.C.); 13009239409@163.com (X.L.)

**Keywords:** *Tetrastichus septentrionalis*, diapause, supercooling point

## Abstract

**Simple Summary:**

*Hyphantria cunea* (Drury) (Lepidoptera: Erebidae), also known as the fall webworm, is a worldwide quarantine pest. In recent years, it has spread and caused serious damage in China. *Tetrastichus septentrionalis* Yang is a native dominant parasitic natural enemy population found in China that has potential application value for biological control. We selected *Tenebrio molitor* as an alternative host for artificially rearing *T. septentrionalis* via screening. We found that diapause in *T. septentrionalis* is of the long-day type, and can also be used in insect enemy mass production. This study’s findings may allow the preservation period of this species to be prolonged by utilizing the phenomenon of diapause in the future and provide a reference for the biological control of the fall webworm and other leaf-eating pests.

**Abstract:**

The chalcid wasp *Tetrastichus septentrionalis* Yang (Hymenoptera: Eulophidae) is one of the dominant pupal parasitoids of *Hyphantria cunea* (Drury) (Lepidoptera: Erebidae). In this study, the photoperiod’s effect on diapause induction in *T. septentrionalis* using the alternative host *Tenebrio molitor* was measured, revealing that *T. septentrionalis* is of the long-day type. The critical photoperiods for diapause induction in *T. septentrionalis* were estimated to be between photoperiods of 13:11 and 14:10 (L:D) h at 18 °C, and between photoperiods of 12:12 and 13:11 (L:D) h at 21 °C and 24 °C. We also found that *T. septentrionalis* diapausing larvae were grey-brown, while normally developed (non-diapausing) individuals were light yellow. The diapause-sensitive insect state was the larval stage, and the short light exposure treatment had a significant cumulative effect on diapause induction. The least squares method was used to calculate a lower developmental threshold of 13.34 ± 0.50 °C and an effective cumulative temperature of 184.46 ± 11.46 d·°C for post-diapause development. The average supercooling point of diapausing mature larvae was significantly lower than that of non-diapausing ones. Our research on *T. septentrionalis* provides a reference for the biological control of *H. cunea* and other leaf-eating pests.

## 1. Introduction

As the most abundant taxon on Earth, insects live in environments that are not constantly stable. Changes in photoperiods, temperature fluctuations, weather extremes, droughts, and food shortages can all threaten their growth and development [1]. Diapause is a strategy adopted by many insects affected by environmental changes to evade adverse weather conditions and maintain the continuation of their populations [2]. Diapause is essential for the survival of insect species, and any disturbance to the time or expression of diapause could have negative consequences. There are, therefore, mechanisms to manage pest control and for their natural enemies to reproduce, but so far, these ideas have rarely been translated into practical control strategies [3]. Diapause is a form of dormancy used by insects to survive adversity and is characterized by reduced metabolic activity and arrested morphological development, which can only be broken by specific environmental stimuli or physiological changes [4]. When the correct stage for diapause has been attained, the insect arrests its development, switches on the new metabolic machinery that will sustain it during metabolic suppression, and then, “decides” the correct time to resume development [5].

Many environmental factors play a crucial role in diapause induction, including temperature and photoperiod. The current research on insect seasonal ecology has focused on temperature and photoperiod as the main regulatory factors [6]. Insects are poikilothermic animals, and high or low ambient temperatures can cause a series of physiological and biochemical reactions in insects, which can then reduce the reproduction of insect populations [7]. Insects have developed adaptations to unfavorable environmental temperatures [8], and insects can improve their cold tolerance by regulating their supercooling points (SCPs), so the supercooling point and freezing point are important indicators used to evaluate insects’ cold tolerance [9]. Low winter temperatures can limit the survival of organisms, particularly those with ectotherm temperatures that are similar to their environment [10]. The cold tolerance of insects was studied in the 18th century, and the discovery of insects’ SCPs provided the basis for practical research into insects’ adaptation to low-temperature environments and many related issues [11]. The SCP is the temperature at which an insect’s body fluid begins to change from liquid to solid. It is the lowest temperature that insects reach before heat is released. This is because the heat released via water crystallization raises the temperature [12].

*Hyphantria cunea* (Drury) (Lepidoptera: Erebidae), also known as the fall webworm, is native to North America and is a worldwide quarantine pest. Different methods have been adopted in China for the biological control of *H. cunea*, but, at present, only *Bacillus thuringiensis* (Bt), nuclear polyhedrosis virus (NPV), and *Chouioa cunea* Yang (Hymenoptera: Eulophidae) are widely used for field management, while the application of *Paenibacillus* spp. and pathogenic nematodes is still in a small or experimental stage [13]. As of 2021, there are 82 species of natural enemies of *H. cunea* in China, including 29 predatory and 53 parasitic natural enemies [14]. Among the existing control technologies for *H. cunea*, population-monitoring technology with sex pheromones and the use of *C. cunea* for the biological control of *H. cunea* have already been implemented [15].

*Tetrastichus septentrionalis* Yang (Hymenoptera: Eulophidae) was reared from fall webworm pupae. This species group is an important pupal parasitoid of lepidopteran pests and can serve as a primary parasitoid for 40 species of lepidopteran pests. *T. septentrionalis* and *C. cunea* were both discovered by Chinese entomologists in the pupae of *H. cunea* in the wild [16]. They are also native Chinese parasitoids that can be used for the biological control of *H. cunea*. *C. cunea*, due to its unique characteristics and extensive research, has been selected as a widely applied biological control agent. *T. septentrionalis* shows great potential and may contribute to the enrichment of parasitoids in the future. Previous experiments have been performed on the biological characteristics, artificial-scale rearing, and field release techniques of *T. septentrionalis*, showing that the parasitoid is the pupal stage of lepidopteran pests. The natural parasitism rate for Lepidoptera is 5–20%, while the parasitism rate for indoor rearing can be significantly increased to 60–70%, and the overall control effect on pests can reach approximately 30% after forest release [17]. *T. septentrionalis* is a common natural enemy of *H. cunea* and other lepidopteran leaf-eating pests during the pupal stage [18].

Our experiment aimed to explore the effects of the photoperiod on diapause in *T. septentrionalis* at different temperatures. We measured and determined the diapause type, the diapause-sensitive developmental stage, the critical diapause photoperiod under different temperature conditions, and the supercooling point (SCP). We selected *Tenebrio molitor* as an alternative host for the artificial rearing of *T. septentrionalis* via screening. The results can be used in insect enemy mass production against *H. cunea* [19]. For example, we can prolong the preservation period of this species by utilizing the phenomenon of diapause in the future.

## 2. Methods

### 2.1. Insect Production

All *T. septentrionalis* specimens used in this study were obtained from laboratory-reared colonies maintained at Shenyang Agricultural University. The populations were the offspring of insects originally collected in *H. cunea* in 2020 from Dandong City, Liaoning Province. The laboratory colony was reared in constant-temperature light incubators (RXZ-260A. Ningbo Dongnan Instrument Co., LTD, Ningbo, China) at 23–26 °C and 60–70% relative humidity (RH) and with a 16:8 (16 h light:8 h dark) photoperiod.

### 2.2. Experiment 1: The Critical Photoperiod for Diapause Induction under Different Temperature Conditions

To determine the critical photoperiod for diapause induction in *T. septentrionalis*, we set three temperatures (18 °C, 21 °C, and 24 °C) and 10 photoperiods (L7:D17, L8:D16, L9:D15, L10:D14, L11:D13, L12:D12, L13:D11, L14:D10, L15:D9, and L16:D8) according to the gradient. A total of 30 treatments were performed in this experiment. During the experiment, the pupae of *T. molitor* 1–2 days after pupation were each put into a 10 mL plastic centrifugation tube. *T. septentrionalis* was inoculated within 48 h of emergence, and the ratio of males was 25%. A skimmed cotton ball dipped in 10% honey water was placed at the mouths of 1/3 of the tubes for the parasitic wasps to replenish their nutrients, and then, the skimmed cotton was sealed. The wasps were removed from the tubes 48 h after the females had laid their eggs. All-black opaque cartons (23 cm × 10 cm × 5 cm) were used to simulate a dark environment, and the pupae of *T. molitor* that had been parasitized by wasps were placed in a constant-light incubator according to the temperature and light combinations described above. Ten replications per combination were treated and replicated three times. The pupae were dissected, and the wasps were observed and counted.

### 2.3. Experiment 2: Morphological Observations of the Diapause State of T. septentrionalis

The differences in color and morphology between diapause and non-diapause larvae were observed using a stereomicroscope (SMZ161, Shenzhen Huaxian Optical Instrument Co., Ltd., Shenzhen, China) at 21 °C.

### 2.4. Experiment 3: Sensitive Insect State Determination for Photoperiod-Induced Diapause

Based on the results of the above experiments (Experiment 1), 21 °C and 14 h of long light (L) and 10 h of short light (S) were chosen as the conditions to determine the sensitive insect state. This experiment was divided into two groups: the first group included pupae of *T. molitor* that had been parasitized by wasps and treated with a short-light (S) period followed by a long-light (L) period (Table: Treatments 1–13); the other group included parasitized pupae of *T. molitor* treated with a long-light (L) period followed by a short-light (S) period (Table: A–M). Each treatment was given with 5 replications and was replicated 3 times. The pupae were observed regularly every day, and the individuals that continued to grow and became dormant under different temperatures and photoperiods were counted.

### 2.5. Experiment 4: Determination of Post-Diapause Developmental Starting Temperatures and Effective Cumulative Temperatures

The mature larvae of *T. septentrionali* were labeled and numbered in black opaque paper boxes at 21 °C and under a 10:14 h light/dark (L:D) photoperiod, and then, placed in a refrigerator at 4 °C for 30 d in total darkness (DD). Immediately afterward, they were removed and transferred to a constant-light incubator at 18 °C, 21 °C, and 24 °C under a 10:14 h light/dark (L:D) photoperiod. Each group was treated with 20 pupae, and the developmental period, the number of emerged wasps, and the ratio of males were recorded. When the wasps started to emerge, the number of emergences was recorded every day. This involved transferring the wasps from a low-temperature refrigerator set at 4 °C and subsequently incubating them at a constant temperature. The observation continued until 50% of the wasps had emerged. The effects of the lower developmental threshold (*C*) and the effective cumulative temperature (*K*) of the post-diapausing development of *T. septentrionalis* were calculated using the ordinary least squares method according to the regression equation of temperature and the developmental rate in the effective cumulative temperature rule, and the standard deviations *S_c_* and *S_k_* were calculated according to Equation [20]. The specific formula is as follows:

$$\begin{array}{l} K=\frac{n\sum VT-\sum V\sum T}{n\sum V^{2}-{\left(\sum V\right)}^{2}}\\ C=\frac{n\sum V^{2}\sum T-\sum V\sum VT}{n\sum V^{2}-{\left(\sum V\right)}^{2}}\\ S_{k}=\sqrt{\frac{\sum {\left(T-T^{\prime }\right)}^{2}}{\left(n-2\right)\sum {\left(V-\bar{V}\right)}^{2}}\left(\frac{1}{n}+\frac{{\bar{V}}^{2}}{\sum {\left(V-\bar{V}\right)}^{2}}\right)}\\ S_{c}=\sqrt{\frac{\sum {\left(T-T^{\prime }\right)}^{2}}{\left(n-2\right)\sum {\left(V-\bar{V}\right)}^{2}}} \end{array}$$

*K* is the effective cumulative temperature constant, *n* is the sample size, *T* is the environmental temperature, *V* is the developmental rate, *C* is the developmental starting temperature, $\bar{V}$ is the mean developmental rate, and *T′* is the theoretical temperature value.

### 2.6. Experiment 5: Measurement of the Supercooling Point and Freezing Point of T. septentrionalis

Diapausing and non-diapausing mature larvae of the same developmental status were selected to determine the supercooling and freezing points. The supercooling point is the lowest body temperature reached before body fluids begin to freeze [21]. The freezing point is the temperature at which liquid freezes in insects. The insects were blotted with filter paper before the measurement, and then, the supercooling point (SCP) and freezing point (FP) were measured using a digital thermometer (UT320, UNI-T, Dongguan, China). During the measurement, each insect body was fixed on the surface of a temperature-sensitive probe using a self-made foam fixing device, and the centrifuge tube was covered to connect the temperature-sensitive probe in contact with the abdomen of the insect body to the temperature detector. The SCP and FP were measured in a temperature-controlled refrigerator at −40 °C. The data were recorded on a computer. The insects were cooled down at a rate of 1 °C/min and the data recorded once per second [22,23]. Measurements of the groups of 20 larvae of diapause and non-diapause *T. septentrionalis* were replicated three times.

### 2.7. Statistical Analyses

The experimental data were analyzed using SPSS 22.0 software. Two-way ANOVA was used to analyze the induced diapause rate of the larvae under different temperatures and photoperiods. Duncan’s new complex polar difference method (*p* < 0.05) was used to test the significance of the difference. The supercooling point and freezing point data were analyzed using an independent samples *t*-test. Diapause rates were calculated as follows:

$$\mathrm{Diapause}\;\mathrm{rate}\;\left(\%\right)=\frac{\mathrm{Number}\;\mathrm{of}\;\mathrm{diapause}\;\mathrm{larvae}}{\mathrm{Total}\;\mathrm{number}\;\mathrm{of}\;\mathrm{insects}\;\mathrm{observed}\left(\mathrm{larvae}+\mathrm{pre}-\mathrm{pupae}+\mathrm{pupae}+\mathrm{adults}\right)}\times 100\%$$

## 3. Results

### 3.1. Experiment 1: The Effect of Photoperiod on T. septentrionalis and Its Critical Photoperiod under Different Temperature Conditions

**Figure 1:**
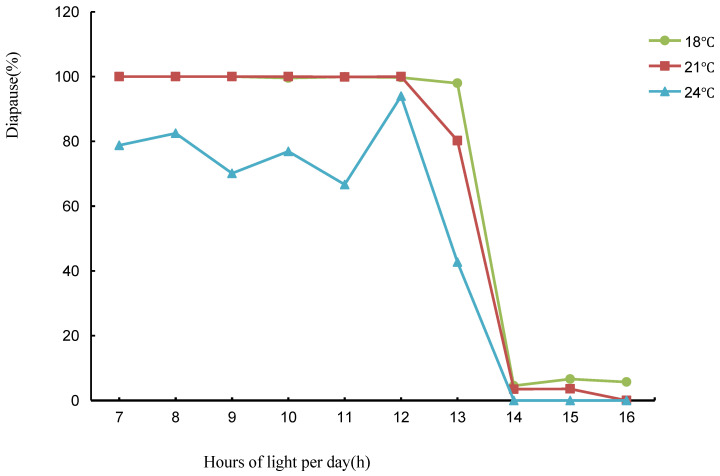
Photoperiodic response of diapause induction in *T. septentrionalis* at 18 °C, 21 °C, and 24 °C.

The photoperiodic response curves of the wasps at different temperatures (Figure 1) showed that the wasps were of the long-day type in the ecological photoperiod range, and the diapause rate of the wasps at different temperatures tended to decrease as the number of light hours (7–16 h) increased. In the range of 7–12 h, the diapause rate was 100% at 18 and 21 °C, and fluctuated but remained above 60% at 24 °C. When the photoperiod was longer than 12 h, the diapause rate decreased significantly with increasing photoperiod. When the photoperiod was 13 h, the diapause rate of wasps showed significant differences in the three temperature conditions. When the photoperiod was longer than 14 h, the diapause rate was close to 0% at all three temperatures. In conclusion, the dominant factor in diapause induction is the duration of light, and this wasp is a typical long-day type.

Although light plays a major role in diapause induction in *T. septentrionalis*, the important role of temperature in diapause induction cannot be ignored. When the photoperiod was less than 12 h or more than 14 h, diapause was mainly regulated by the photoperiod, and temperature had little or no effect on diapause, but when the photoperiod exceeded 12 h, the diapause rate decreased with increasing temperature. The differences between the different temperature treatments were significant (Table 1). These results show that lower temperatures can promote the occurrence of diapause in *T. septentrionalis*, while higher temperatures have an inhibiting effect on diapause.

According to the photoperiodic response curves and the diapause rate of the wasps at different temperatures in Table 1, the critical photoperiod to induce diapause was between 13 h and 14 h at 18 °C. At 21 °C and 24 °C, the critical photoperiod to induce diapause was shorter than at 18 °C, between 12 h and 13 h, and the critical photoperiod increased as the temperature decreased. In addition, almost all individuals developed normally under a 14–16 h photoperiod. At 18 °C and 21 °C, 100% of individuals were induced to diapause when the photoperiod was less than or equal to 12 h. At 24 °C, most individuals were induced to diapause when the photoperiod was less than or equal to 12 h, but the diapause rate was slightly lower than in the other two groups.

In Figure 1, a significant correlation was found between the diapause rate and photoperiod for *T. septentrionalis.* Therefore, the model was fitted using SPSS software to obtain the best-fitting equation for the diapause rate and the photoperiod of *T. septentrionalis* at 12–14 h. This was used to calculate the critical photoperiod (X: the photoperiod required for 50% of individuals to enter diapause; Y: the diapause rate at different temperatures). The quadratic function was found to be the best fit. When Y = 0.5, the critical photoperiods of *T. septentrionalis* at 18, 21, and 24 °C were L:D = 14 h 2 min:9 h 58 min, L:D = 14 h 2 min:9 h 58 min, L:D = 14 h 2 min:9 h 58 min, L:D = 14 h 2 min:9 h 58 min, and L:D = 14 h 0 min:10 h 0 min (Table 2).

### 3.2. Experiment 2: Comparison between the Diapause and Non-Diapause Insect Morphology of T. septentrionalis

**Figure 2:**
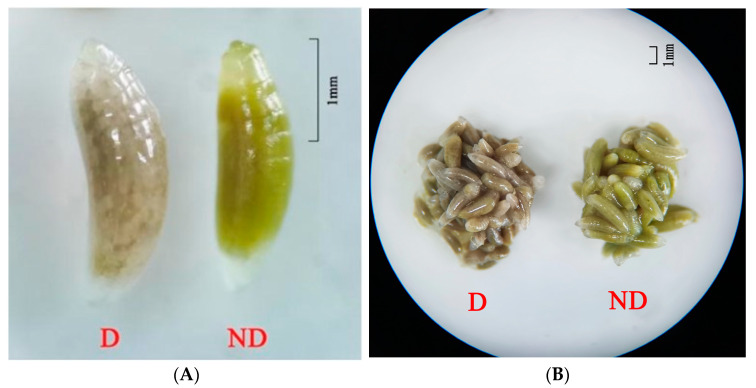
Morphological characteristics of diapause versus non-diapause larvae of *T. septentrionalis.* Note: (**A**) shows a comparison between individual larvae, and (**B**) shows a comparison between groups.

Under laboratory conditions, the non-diapause mature larvae of *T. septentrionalis* were light yellow, spindle-shaped, had inconspicuous subcutaneous fat particles, and were able to pupate within a short period (Figure 2, ND). Conversely, the mature larvae of *T. septentrionalis* treated with a low temperature and short light period were grey-brown, stout, had conspicuous subcutaneous fat particles, and remained in the diapause state for a long time (Figure 2, D). Therefore, the color and morphological characteristics of mature larvae can be used as a basis for identification.

### 3.3. Experiment 3: Photoperiod-Induced Diapause Induction of Sensitive Insect States

As shown in Table 3, (1) the larval stage was the sensitive state for diapause (3–13, A–H), while the egg and pupal stages did not affect diapause whether they received the short-light treatment (1–2, I–M) or long-light treatment (A–B, 9–10), and (2) there was no significant relationship between the diapause rate and the pre-larval and post-larval stages in the short-light treatment. The diapause rate in the larval stage was proportional to the number of days receiving the short-light treatment. The short-light-exposure treatment had a significant cumulative effect on diapause induction in *T. septentrionalis*. The diapause rate approached 100% when the larval period was treated with short light for more than 8 d at 21 °C, and the diapause rate of the wasps reached 100% when the entire larval period received the short-light treatment (A–B, 9–13).

### 3.4. Experiment 4: Post-Diapause Developmental Starting Temperatures and Effective Cumulative Temperatures of T. septentrionalis

The post-diapause developmental periods of *T. septentrionalis* were 35.42 d, 26.75 d, and 17.00 d at constant temperatures of 18 °C, 21 °C, and 24 °C, respectively. The differences between temperature gradients were significant (*p* < 0.05). Linear regression was carried out based on the rate of post-diapause development at different temperatures (the regression equation was Y = 0.005X − 0.066, R^2^ = 0.942), and the least squares method was used to calculate a starting temperature of 13.34 ± 0.50 °C and an effective cumulative temperature of 184.46 ± 11.46 d·°C for post-diapause development (Table 4).

### 3.5. Experiment 5: Comparison of the Supercooling Point and Freezing Point between Diapause and Non-Diapause T. septentrionalis

There were differences in the supercooling and freezing points of diapause and non-diapause larvae of *T. septentrionalis* under different temperature conditions. The larvae of the diapause state had the lowest values at 18 °C (−23.26 ± 0.25) °C and (−20.87 ± 0.30) °C, followed by 21 °C (−22.09 ± 0.31) °C and (−19.90 ± 0.31) °C, and the highest at 24 °C (−18.69 ± 0.37) °C and (−15.49 ± 0.42) °C, respectively. Similarly, there were significant differences between the supercooling and freezing points of non-diapause larvae at the three different temperatures, with the lowest supercooling and freezing points at 21 °C (−20.20 ± 0.42) °C and (−17.26 ± 0.47) °C, respectively, followed by 18 °C (−19.51 ± 0.48) °C and (−13.84 ± 0.55) °C, and the highest at 24 °C (−15.49 ± 0.42) °C and (−10.25 ± 0.42) °C, respectively (Table 5).

**Figure 3:**
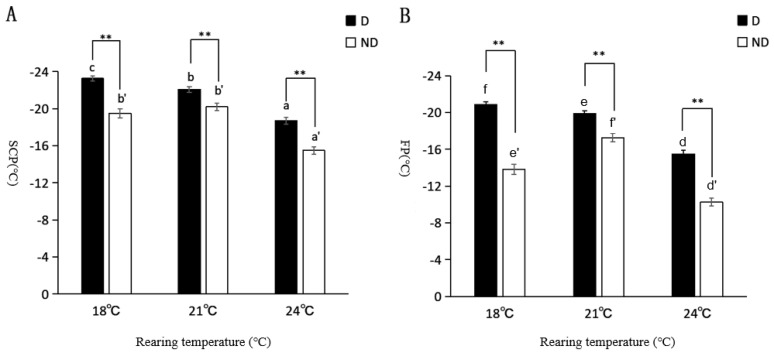
Supercooling points (SCPs) and freezing points (FPs) of diapause and non-diapause larvae of *T. septentrionalis* at different temperatures. Note: (**A**) shows the supercooling points of *T. septentrionalis*, and (**B**) shows the freezing points of *T. septentrionalis*. Lowercase letters indicate a significant difference between supercooling points and freezing points at different temperatures in the same specific state. ** indicates a highly significant (*p* < 0.01) difference in supercooling points and freezing points between different insect states under the same temperature conditions. D: diapause; ND: non-diapause. a, b, and c represent the differences in SCP of diapause larvae of *T. septentrionalis* at different temperatures. a′, b′ and c′ represent the differences in SCP of non-diapause larvae of *T. septentrionalis* at different temperatures. d, e, and f represent the differences in FP of diapause larvae of *T. septentrionalis* at different temperatures. d′, e′ and f′ represent the differences in FP of non-diapause larvae of *T. septentrionalis* at different temperatures.

The results in Figure 3 show that the supercooling point and freezing point of the larvae of *T. septentrionalis* increased with increasing temperature, while the supercooling point and freezing point of non-diapause larvae showed an increasing, and then, a decreasing trend with increasing temperature. The supercooling point and freezing point of the diapause larvae at 18 °C were significantly lower than those at 21 °C and 24 °C under the three different temperature conditions, with significant differences (*p* < 0.05). The supercooling point of the non-diapause larvae was lower at 21 °C compared with that at 18 °C and 24 °C. The difference between the supercooling point and freezing point of the larvae of the two insect forms was highly significant (*p* < 0.01) at the same temperature.

## 4. Discussion

Dandong is located in the northern part of China, where the average winter temperature is approximately −6 °C, and the lowest temperature can reach approximately −20 °C in winter. Therefore, insects in this area generally have strong cold resistance in winter. Therefore, we designed an experiment that demonstrated that *T. septentrionalis* could enter diapause. Our team conducted the experiment of screening the best alternative host of *T. septentrionalis* among the pupae of *H. cunea*, *Antherea pernyi*, *T. molitor*, and *Zophobas atratus*. The results showed that *T. molitor* is a better alternative host and can be obtained at any time with a low cost [19]. In the future, *T. molitor* will be used to rear *T. septentrionalis* for biological control, so *T. septentrionalis* reared by *T. molitor* was used for this study. As a pupal parasitic natural enemy of pests, studying this species’ diapause in alternative hosts can lay a foundation for the biological control of artificial reproduction. For example, the diapause characteristics of *T. septentrionalis* can prolong the preservation period in mass production. *C. cunea* for biological control is reared using *A. pernyi* instead of *H. cunea* in China. Therefore, in the diapause experiment of *C. cunea*, *A. pernyi* was used as an alternative host for rearing [24]. The diapause rate of *T. septentrionalis* varied with increasing temperature and photoperiod. Temperature is considered an important factor that influences dormancy in several ways: (1) temperature is a major diapause-inducing factor in some species; (2) it can modify insects’ response to diapause-inducing photoperiods to varying degrees; (3) in some species, it is important for diapause maintenance; and (4) it can be an active stimulus for the termination of diapause [25]. The developmental period was significantly shorter, and the developmental rate was significantly faster, with increasing temperature. In our previous study on the influence of temperature and the photoperiod on diapause regulation, the results also confirmed that temperature and photoperiod are critical for diapause induction in *C. cunea* [24]. Temperature had a significant effect on the post-diapause development of *T. septentrionalis*. The developmental rate was accelerated with increasing temperature, and the post-developmental time was significantly shortened.

Beck proposes two types of response curves, long-day and short-day. The long-day type grows, develops, and reproduces under long-daylight conditions and enters diapause for a short period. The short-day type is less common and is shown in the response of summer-dormant insects to long-daylight conditions [26]. Under the same temperature conditions, the diapause rate of *T. septentrionalis* with more than 16 h of light was zero percent. Conversely, the diapause rate of *T. septentrionalis* with less than 12 h of light was approximately one hundred percent. *T. septentrionalis* is a long-day type. A short photoperiod has a cumulative effect on diapause induction in *T. septentrionalis*, and the larval stage in a short photoperiod is the stage most responsive to photoperiodic stimuli. *T. septentrionalis* is sensitive to light throughout the larval period, and the photoperiod plays a decisive role in the diapause process, while temperature plays an assistant role. A low temperature is conducive to an increase in the larval diapause rate. Similar results have been found in related studies, for instance, *C. cunea* and *Anastatus janponicus* both diapause as mature larvae, and short-day and low-temperature conditions are the main factors that induce them to diapause [24,27].

The diapause and non-diapause larvae were significantly different in color, with the non-diapause larvae being light yellow, while the diapause larvae were grey-brown. *C. cunea* is another important pupal parasitic natural enemy of *H. cunea*. The color of the diapause-stage larvae of *C. cunea* also exhibited similar changes. The body color of the diapausing *C. cunea* larvae was taupe, while the normally developed larvae were light yellow. Therefore, this body color change can be used as an indicator of diapause entry in insects. Color differences between insects developing directly and those in diapause have often been used to distinguish between these two developmental stages [28,29,30]. Other insect studies have also found that the color of the diapause state changes during diapause. In *Byasa alcinous*, the color of diapausing pupae in the wild was dark brown, while they appeared brown, light brown, yellowish-brown, light yellow, and yellow in an indoor environment [31]. In *Sericinus montelus*, the non-diapause pupae were light yellow, while the diapause pupae were darker than the non-diapause pupae. [32]. To explain the cause of this color change, the research on *B. alcinous* suggests that temperature and humidity are the main factors that affect diapause pupal coloration as environmental cues [31]. An experiment in *Nezara viridula* showed that changes in adult color are gradual and controlled by photoperiodic conditions because adults remain green under long-day conditions and turn russet under short-day conditions [33]. However, not all color changes in diapause insects are related to diapause; these changes may also be responses to cold hardiness, desiccation resistance, or camouflage in changed habitats. The reasons for the color changes in diapause and non-diapause insects need to be further studied.

The diapause-sensitive stage is the developmental stage in which an insect can be influenced by diapause induction factors during its life history [34]. It is constant for a particular insect, but there are significant differences between an insect’s diapause and diapause-sensitive states [26]. The sensitive stage of *T. septentrionalis* comprises the whole larval period. A short photoperiod has a cumulative effect on diapause induction in *T. septentrionalis*, and the larval stage is more conducive to diapause. The results of this study are consistent with the results of diapause in *C. cunea*, where the sensitive stage of diapause is also the larval stage [24]. *Aphidius gifuensis* diapause as pupae and mature larvae, and their second–third instars are sensitive to diapause signals, and third-instar larvae are more sensitive to diapause induction signals [35,36]. *M. mediator* diapause as pupae and are sensitive to second-stage juveniles [37]. As parasitoids have different insect stages for diapause, the sensitive insect stages for diapause also vary between different insects. *Trichogramma evanescens*, *Trichogramma embryophagum*, *Trichogramma principium*, and *Trichogramma minutum* are pre-pupal diapause, and the sensitive stage of diapause is the larval stage [38,39,40,41]. *Hoclcothorax testaceipes* and *Psyllaephaqus pistaciae* diapause in the pupal stage, and the sensitive stage of diapause is from the mature larval to the pre-pupal stage [42,43]. *Trichogramma brassicae* diapause can be induced by starting with the egg stage, pre-larval stage, and middle larval stage [44,45,46].

Current research has found that some insects also experience mass mortality at sublethal temperatures above their supercooling points, but insects’ supercooling ability has a significant role in determining their cold tolerance [47]. In this experiment, we measured the supercooling point and freezing point, which can provide a basis for the cold tolerance of *T. septentrionalis*. *T. septentrionalis* overwintered in the pupae of *H. cunea* as mature larvae. The overwintering insects were exposed to temperatures that would freeze them to death. According to our determination of the cold tolerance of *T. septentrionalis*, the supercooling point and freezing point of the diapause larvae were significantly lower than those of the non-diapause larvae at different temperatures. The results of the cold tolerance assay showed that both the supercooling point and freezing point in diapause larvae increased with increasing incubation temperature. Considering an insect’s supercooling point as a cold tolerance indicator, diapause induction can effectively improve the cold tolerance of larvae and help them adapt to the cold environment in winter. There are three measures commonly used to assess cold tolerance at the population level in insects: the supercooling point, lower lethal temperature, and lower lethal time [48,49]. In future experiments, the study of lower lethal temperatures and lower lethal times will provide a direction for further research on cold tolerance. Similar results were found in our study on *C. cunea* [24]. As an important pupal parasitoid natural enemy of *H. cunea* in Liaoning Province, studying the cold tolerance of *T. septentrionalis* can provide a basis for determining whether its natural population can spread further north.

In this study, the critical photoperiod of diapause was found at different temperatures, and the diapause state, sensitive state, supercooling point, and freezing point were observed and measured. This research on the diapause and cold tolerance of *T. septentrionalis*, an important parasitoid natural enemy of *H. cunea*, can provide theoretical and technical support for extending the pest control time and improving the stress resistance and reproductive ability of this wasp.

## Figures and Tables

**Table 1:** Effects of temperature and photoperiod on diapause in *T. septentrionalis*.

Photoperiod (L:D)	Actual Measurement Sample No.	Diapause Rate (%) (18 °C)	Diapause Rate (%) (21 °C)	Diapause Rate (%) (24 °C)
7:17	85	100.00 ± 0.00 ^aA^	99.97 ±0.06 ^aA^	97.08 ± 1.87 ^aA^
8:16	89	100.00 ± 0.00 ^aA^	100.00 ±0.00 ^aA^	82.50 ± 7.12 ^bB^
9:15	87	100.00 ± 0.00 ^aA^	100.00 ±0.00 ^aA^	70.12 ± 7.81 ^bB^
10:14	89	99.54 ± 0.46 ^aA^	100.00 ±0.00 ^aA^	76.90 ± 7.68 ^bB^
11:13	89	99.92 ± 0.08 ^aA^	99.89 ± 0.11 ^aA^	66.67 ± 8.75 ^bB^
12:12	89	99.72 ± 0.23 ^aA^	100.00 ± 0.00 ^aA^	93.96 ± 3.93 ^aA^
13:11	89	97.95 ± 1.25 ^aA^	80.23 ± 7.31 ^bB^	42.72 ± 8.95 ^bC^
14:10	87	4.55 ± 3.72 ^bA^	3.50 ± 3.45 ^cA^	0.00 ± 0.00 ^cB^
15:9	83	6.61 ± 3.98 ^bA^	3.57 ± 3.57 ^cA^	0.00 ± 0.00 ^cB^
16:8	88	0.00 ± 0.00 ^cA^	0.00 ± 0.00 ^dA^	0.00 ± 0.00 ^cA^

Note: Data are means ± SE. Different lowercase letters in the same column or different uppercase letters in the same row indicate significant differences at *p* < 0.05 level using Duncan’s new multiple range test.

**Table 2:** Critical photoperiod of *T. septentrionalis* at different temperatures.

Temperature (°C)	Fitted Equation	Coefficient of Determination (R^2^)	Critical Photoperiod
18	Y = −45.815X^2^ + 1143.605X − 7026.18	1.00	L:D =14 h 2 min:9 h 58 min
21	Y = −28.480X^2^ + 692.230X − 4105.640	1.00	L:D = 14 h 2 min:9 h 58 min
24	Y = 4.260X^2^ − 157.740X + 1373.400	1.00	L:D = 14 h 0 min:10 h 00 min

**Table 3:** The diapause rate under photoperiods of 10L:14D and 14L:10D at different developmental stages in *T. septentrionalis* at 21 °C.

Treatment	Approximate Stage													(Mean ± SE) Diapause Rate
	Egg		Larval							Pupal				
	0–2 d	2–4 d	0–2 d	2–4 d	4–6 d	6–8 d	8–10 d	10–12 d	12–14 d	0–2 d	2–4 d	4–6 d	6–8 d	
1	S	L	L	L	L	L	L	L	L	L	L	L	L	0.00 ± 0.00% c
2	S	S	L	L	L	L	L	L	L	L	L	L	L	0.00 ± 0.00% c
3	S	S	S	L	L	L	L	L	L	L	L	L	L	0.11 ± 0.11% c
4	S	S	S	S	L	L	L	L	L	L	L	L	L	1.14 ± 0.57% c
5	S	S	S	S	S	L	L	L	L	L	L	L	L	15.08 ± 5.02% b
6	S	S	S	S	S	S	L	L	L	L	L	L	L	16.28 ± 7.92% b
7	S	S	S	S	S	S	S	L	L	L	L	L	L	98.66 ± 1.11% a
8	S	S	S	S	S	S	S	S	L	L	L	L	L	99.30 ± 0.70% a
9	S	S	S	S	S	S	S	S	S	L	L	L	L	100.00 ± 0.00% a
10	S	S	S	S	S	S	S	S	S	S	L	L	L	100.00 ± 0.00% a
11	S	S	S	S	S	S	S	S	S	S	S	L	L	100.00 ± 0.00% a
12	S	S	S	S	S	S	S	S	S	S	S	S	L	100.00 ± 0.00% a
13	S	S	S	S	S	S	S	S	S	S	S	S	S	99.66 ± 0.34% a
A	L	S	S	S	S	S	S	S	S	S	S	S	S	100.00 ± 0.00% a
B	L	L	S	S	S	S	S	S	S	S	S	S	S	100.00 ± 0.00% a
C	L	L	L	S	S	S	S	S	S	S	S	S	S	99.26 ± 0.74% a
D	L	L	L	L	S	S	S	S	S	S	S	S	S	100.00 ± 0.00% a
E	L	L	L	L	L	S	S	S	S	S	S	S	S	62.19 ± 14.60% b
F	L	L	L	L	L	L	S	S	S	S	S	S	S	16.31 ± 8.48% c
G	L	L	L	L	L	L	L	S	S	S	S	S	S	1.26 ± 0.65% d
H	L	L	L	L	L	L	L	L	S	S	S	S	S	0.72 ± 0.58% d
I	L	L	L	L	L	L	L	L	L	S	S	S	S	0.00 ± 0.00% d
J	L	L	L	L	L	L	L	L	L	L	S	S	S	0.00 ± 0.00% d
K	L	L	L	L	L	L	L	L	L	L	L	S	S	0.00 ± 0.00% d
L	L	L	L	L	L	L	L	L	L	L	L	L	S	0.00 ± 0.00% d
M	L	L	L	L	L	L	L	L	L	L	L	L	L	0.00 ± 0.00% d

Note: S, short-light treatment (10L:14D); L, long-light treatment (14L:10D). Each cell indicates a developmental stage of parasitoid wasps for 2 days, and the intersection of S and L in each row indicates that the short-light treatment was converted to the long-light treatment. Diapause rates are means ± SE, and different letters after the data denote Duncan’s new complex polarization method for significant differences in multiple comparisons (*p* < 0.05).

**Table 4:** Effect of temperature on post-diapause development of *T. septentrionalis*.

Temperature (°C)	Developmental Duration (d)	Developmental Rate (1/d)
18	35.42 ± 0.20 ^a^	0.03 ± 0.02 ^c′^
21	26.75 ± 0.17 ^b^	0.04 ± 0.02 ^b′^
24	17.00 ± 0.22 ^c^	0.06 ± 0.08 ^a′^

Note: a, b and c represent the differences in developmental duration of *T. septentrionalis* at different temperatures. a′, b′ and c′ represent the differences in developmental rate of *T. septentrionalis* at different temperatures.

**Table 5:** Supercooling points and freezing points of diapause and non-diapause larvae of *T. septentrionalis*.

Temperature (°C)	Number of Individuals	Different Treatments of Insect States			
		Diapause		Non-Diapause	
		SCP (°C)	FP (°C)	SCP (°C)	FP (°C)
18	60	−23.26 ± 0.2ff5 ^cB^	−20.87 ± 0.3ff0 ^cB^	−19.51 ± 0.4ff8 ^bA^	−13.84 ± 0.5ff5 ^bA^
21	60	−22.09 ± 0.3ff1 ^bB^	−19.90 ± 0.3ff1 ^bB^	−20.20 ± 0.4ff2 ^bA^	−17.26 ± 0.4ff7 ^cA^
24	60	−18.69 ± 0.3ff7 ^aB^	−15.49 ± 0.4ff2 ^aB^	−15.49 ± 0.4ff2 ^aA^	−10.25 ± 0.4ff2 ^aA^

Note: Data are means ± SE. Different lowercase letters in the same column indicate significant differences at *p* < 0.05 level using Duncan’s new multiple range test. Different lowercase letters in the same row indicate significant differences at *p* < 0.05 level using independent samples *t*-test.

## Data Availability

The data presented in this study are available in this article.
